# An evolutionary perspective on Elovl5 fatty acid elongase: comparison of Northern pike and duplicated paralogs from Atlantic salmon

**DOI:** 10.1186/1471-2148-13-85

**Published:** 2013-04-19

**Authors:** Greta Carmona-Antoñanzas, Douglas R Tocher, John B Taggart, Michael J Leaver

**Affiliations:** 1 Institute of Aquaculture, School of Natural Sciences, University of Stirling, Stirling, Scotland, FK9 4LA, UK

**Keywords:** Atlantic salmon, Elongase of very long-chain fatty acids, Northern pike, Paralogous genes, Whole-genome duplication

## Abstract

**Background:**

The ability to produce physiologically critical LC-PUFA from dietary fatty acids differs greatly among teleost species, and is dependent on the possession and expression of fatty acyl desaturase and elongase genes. Atlantic salmon, as a result of a recently duplicated genome, have more of these enzymes than other fish. Recent phylogenetic studies show that Northern pike represents the closest extant relative of the preduplicated ancestral salmonid. Here we characterise a pike fatty acyl elongase, *elovl5,* and compare it to Atlantic salmon *elovl5a* and *elovl5b* duplicates.

**Results:**

Phylogenetic analyses show that Atlantic salmon paralogs are evolving symmetrically, and they have been retained in the genome by purifying selection. Heterologous expression in yeast showed that Northern pike Elovl5 activity is indistinguishable from that of the salmon paralogs, efficiently elongating C18 and C20 substrates. However, in contrast to salmon, pike *elovl5* was predominantly expressed in brain with negligible expression in liver and intestine.

**Conclusions:**

We suggest that the predominant expression of Elovl5b in salmon liver and Elovl5a in salmon intestine is an adaptation, enabled by genome duplication, to a diet rich in terrestrial invertebrates which are relatively poor in LC-PUFA. Pike have retained an ancestral expression profile which supports the maintenance of PUFA in the brain but, due to a highly piscivorous LC-PUFA-rich diet, is not required in liver and intestine. Thus, the characterisation of *elovl5* in Northern pike provides insights into the evolutionary divergence of duplicated genes, and the ecological adaptations of salmonids which have enabled colonisation of nutrient poor freshwaters.

## Background

Atlantic salmon (*Salmo salar*) have been the focus of considerable research effort as a result of their widespread environmental and economic importance as a sporting and cultured species. In addition, in common with all other salmonids, they possess a comparatively recently duplicated genome, believed to have arisen as a result of a relatively recent autotetraploidisation event between 25 and 100 mya [1,2]. Whole-genome duplication (WGD) has been argued as a powerful evolutionary force creating new raw material for evolution to act upon [3], thus enabling the divergence and neo- or subfunctionalisation of duplicated loci promoting adaptation and speciation. The imprints of three or four ancient duplications can be detected in vertebrate genomes, including a specific event early in teleost evolution and the recent one in salmonids [4,5]. Esocids (members of the pike family) are regarded as having the closest extant preduplicated (diploid) genomes to salmonids, based on molecular phylogenetic studies [6,7], karyotype data [8], and comparative analyses of expressed gene sequences [9,10]. Therefore, Northern pike (*Esox lucius*) is representative of a sister-group to salmonids, and can be viewed as an appropriate species to study the consequences of genome duplication in salmonids. Despite their shared ancestry and overlapping habitats, Atlantic salmon and Northern pike have differing life histories and feeding behaviours where, in freshwaters, pike have a largely piscivorous diet, and salmon a diet rich in terrestrial insects [11]. These differences may be reflected in differing nutritional physiology and, in particular, lipid biochemistry.

Teleosts, like all vertebrates, are unable to synthesise polyunsaturated fatty acids (PUFA) *de novo*, and so they are essential and required in the diet [12,13]. However, which PUFA can satisfy the dietary requirement for essential fatty acids (EFA) varies with species. The long-chain PUFA (LC-PUFA), arachidonic acid (20:4n-6, ARA), eicosapentaenoic acid (20:5n-3, EPA), and docosahexaenoic acid (22:6n-3, DHA), which have essential functions in vertebrate immune defense systems and neuronal membranes [14-16] can be produced endogenously, in some but not all vertebrates, from the base EFA, α-linolenic acid (18:3n-3, ALA) and linoleic acid (18:2n-6, LA), by desaturation and elongation [17]. The capability to produce LC-PUFA from EFA varies between fish species [13,18], and salmonids, including Atlantic salmon, brown trout (*Salmo trutta*), and Arctic charr (*Salvelinus alpinus*) have substantially higher LC-PUFA biosynthetic efficiency in comparison with other freshwater species, including zebrafish (*Danio rerio*), Nile tilapia (*Oreochromis niloticus*) [11,19,20], and Northern Pike [21-24]. The ability to produce LC-PUFA is dependent on the possession and expression of fatty acyl desaturase (*fad*) and fatty acid elongase (*elovl*) genes, and salmonids, in contrast to many other fish species examined, have a complete set of genes and expressed enzymes required for the production of ARA from LA, and EPA and DHA from ALA [25,26]. In Atlantic salmon some of these LC-PUFA biosynthetic enzymes appear to have arisen from duplicated genes, and the subsequent neo- or subfunctionalisation has been hypothesised as an enabling adaptation for salmonids to thrive in relatively nutrient-poor freshwater environments [27].

The aim of the present study was to characterise a critical gene and enzyme of LC-PUFA biosynthesis, Elovl5, in Northern pike and to compare with previously identified, duplicated *elovl5* paralogs in Atlantic salmon. Elovl5 catalyses the first and second elongations of LA and ALA and is therefore essential for the production of LC-PUFA. Thus, comparison between the sequence, activity and expression of pike and salmon Elovl5 genes may provide insights into mechanisms that have driven the evolution and ecological adaptations of salmonids.

## Results

### Northern pike Elovl5 sequence and phylogenetics

**Figure 1:**
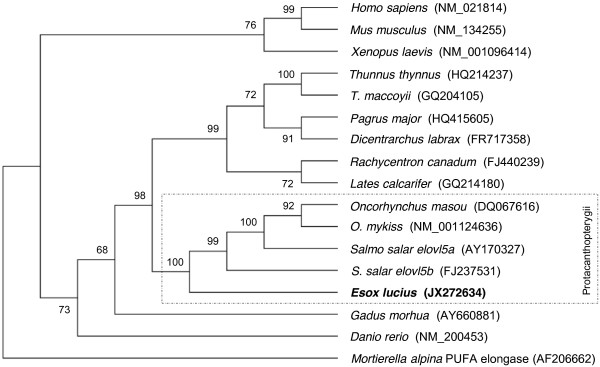
**Phylogenetic tree revealing the relative position of Northern pike Elovl5 according to proteins from other vertebrate orthologs, and rooted to *Mortierella alpina *PUFA elongase as outgroup.** The tree was constructed on the DNA sequences extracted from GenBank using MEGA4 and applying the Neighbour Joining method. The numbers on the branches represent the frequencies (%) with which the presented tree topology was obtained after bootstrapping (10,000 iterations).

A 1,434 bp full-length cDNA sequence (5’UTR 72 bp, ORF 888 bp, 3’UTR 474 bp) was obtained by 5’ and 3’ RACE PCR and submitted to the GenBank database under the accession number JX272634. The pike Elovl5 open reading frame (ORF) encodes a putative protein of 295 AA that shares 69.7% to 71% AA identity to mammalian and reptilian orthologues including human [GenBank:NM_021814], mouse [GenBank:NM_134255] and the frog *Xenopus laevis* [GenBank:NM_00109614]. Phylogenetic analysis shows that teleost *elovl5* genes cluster according to accepted taxonomy as displayed in the phylogenetic tree (Figure 1), with Protacanthopterygii including Salmoniformes and Esociformes forming a clade and thus in agreement with phylogenetic analysis performed upon whole mitochondrial genomes [9]. Among all teleosts, pike exhibit the highest amino acid identity scores with the salmonid Elovl5 members, with Atlantic salmon Elovl5a and Elovl5b being the most similar (86.4%) and dissimilar (83.4%), respectively. Lower identity values were observed in comparison with Elovl5 sequences of species belonging to orders other than Salmoniformes ranging from 73% (*Gadus morhua*) to 80% (*Lates calcarifer*). All fish *elovl5* grouped together with reptilian and mammalian homologs, and more distantly from other members of the *elovl* family (not included in the phylogenetic tree, [28]).

**Figure 2:**
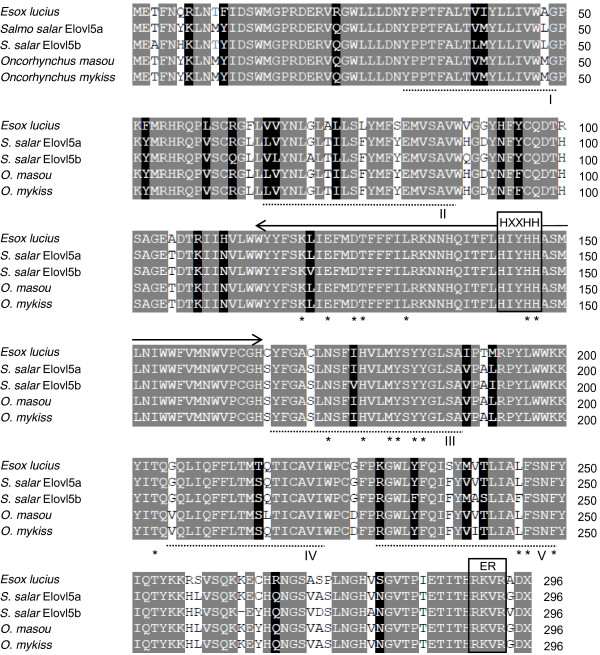
**Alignment of the deduced amino acid sequence of the Northern pike Elovl5 with orthologs of other members of the Protacanthopterygii lineage.** The amino acid alignment was performed using the BLOSUM62 matrix from BioEdit, and identity/similarity was calculated based on a 60% identity threshold. Identical residues are shaded in grey, and altered residues are shaded in white if they exhibit the same chemical qualities, or black if they do not. Outlined are the HXXHH histidine box, and the endoplasmic reticulum retention signal (ER); the five putative transmembrane domains (I-V) are dash-underlined; the predicted “catalytic site” is indicated by the solid arrow above the sequences; and an asterisk indicates each of the 17 amino acid residues conserved across Elovl proteins [31].

The pike Elovl5 deduced amino acid sequence contains the three typical features present in all Elovl members: a single HXXHH histidine box motif, a carboxyl-terminal targeting signal responsible for the retention of transmembrane protein to the endoplasmic reticulum (ER), and multiple putative transmembrane-spanning domains containing hydrophobic AA stretches. The best hydrophobicity model predicted 5 transmembrane helices (transmembrane domain ≥ 20 AA) in accordance with previous analysis using the GES algorithm [29,30]. However, these two methods compute protein polarity scores based upon different chemical arguments resulting in slightly different transmembrane boundaries (± 2 AA). Thus, for greater reliability the transmembrane domains depicted in Figure 2 represent the overlapping regions described by both methods. Additionally, 16 out of the 17 AA residues that have been established to be highly conserved across 22 members of the Elovl family [31] were identified in all protacanthopterygian Elovl5 proteins.

### Purifying selection on salmonid Elovl5 paralogs

**Figure 3:**
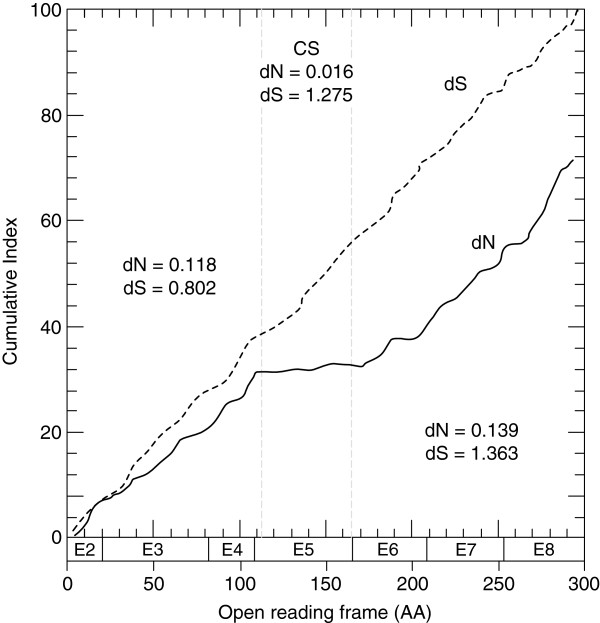
**Average synonymous and nonsynonymous substitutions in vertebrate Elovl5 orthologs.** Cumulative index of the synonymous (dS, dashed line) and nonsynonymous (dN, solid line) substitutions for all the pairwise comparisons are plotted against the open reading frame of vertebrate Elovl5 orthologs. SNAP was used under the default settings to compute the overall dS and dN for all the pairwise comparisons in the predicted “catalytic site” and flanking areas. CS, indicates the region of the predicted catalytic site. Exon boundaries are depicted along the X-axis, E2-E8.

The number of synonymous (dS) and nonsynonymous substitutions (dN) per site was determined by comparing the Northern pike ORF sequence to each of the duplicate Atlantic salmon ORF, and the salmon duplicate ORF to each other. The selection tests indicated that negative (purifying) selection was the major evolutionary force acting on the salmon duplicates since their divergence from Northern pike, with ω equal to 0.24, or 0.20 when using the GA-branch (dN > dS, *P* < 0.01), or SNAP (dN = 0.064, dS = 0.330) approaches, respectively. Accordingly, the average ω between all vertebrate members included in the phylogenetic tree also confirmed overall purifying selection (ω < 1) (GA-branch, *P* < 0.01; SNAP, dN = 0.178, dS = 1.434). When salmon paralogs were compared to one another using pike Elovl5 amino acid sequence as the outgroup the results indicated that both duplicates exhibit comparable evolutionary rates with molecular clock-like behaviour (χ^2^ = 3.56, *P* > 0.05). In contrast, the results obtained when Tajima’s test was performed on the nucleotide data of the aforementioned sequences showed that the salmon *elovl5* sequences are evolving asymmetrically (χ^2^ = 6.75, *P* < 0.05). This suggested that, despite the fact that the salmon elongases appear to be subjected to functional constraints in order to maintain the functionality of the protein, the nucleotide sequence of one of the duplicates seems to be diverging faster than the other. The ORF sequences of the vertebrate *elovl5* members included in this study were codon-aligned, and the accumulated dN and dS substitutions along the coding sequence assessed using SNAP. Results revealed substantial differences in dN throughout the protein sequence. A region corresponding with exon 5 (109–165 AA), which includes the catalytic histidine box, displayed 7 to 8-fold reduction in nonsynonymous substitutions with respect to the flanking regions (Figure 3).These results indicate that selective pressure is not constant along the coding sequence.

### Functional characterisation of pike Elovl5

**Figure 4:**
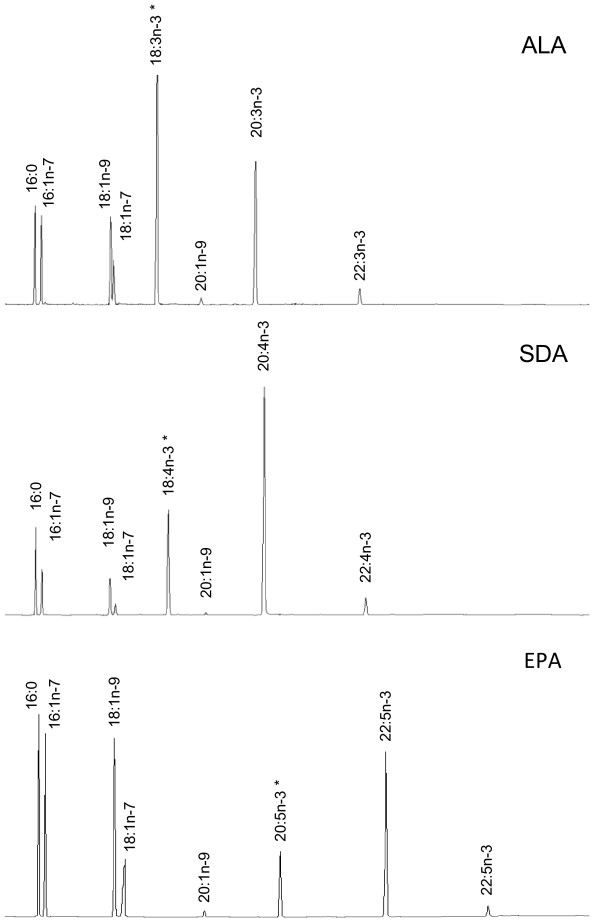
**Identification of fatty acid elongation products in transgenic yeast ( *S. cerevisiae *) transformed with pike Elovl5 ORF and grown in the presence of LC-PUFA substrates.** Yeast were incubated in presence of PUFA substrates α-linolenic acid (ALA, 18:3n-3), stearidonic acid (SDA, 18:4n-3), or eicosapentaenoic acid (EPA, 20:5n-3), and fatty acid elongation products determined. Each panel shows the main endogenous fatty acids of *S. cerevisiae*, namely 16:0, 18:0, 16:1n-7 and 18:1n-9, and their elongated products 18:1n-7 and 20:1n-9. * Substrates and their corresponding elongated products are indicated accordingly in the panels. Vertical axis, FID response; horizontal axis, retention time.

The ability of Northern pike Elovl5 to elongate LC-PUFA of the omega-3 and omega-6 series was determined by the relative quantification of the fatty acid conversions obtained when transformed *Saccharomyces cerevisiae* containing either the empty pYES2 vector (control), or a vector with the pike Elovl5 ORF insert was grown in presence of potential PUFA substrates. Yeast cultures transformed with pYES2 containing the pike Elovl5 ORF and grown in the absence of PUFA substrates showed that pike Elovl5 is capable of efficiently converting the yeast endogenous monounsaturated fatty acids 16:1n-7 and 18:1n-9 to their elongated products determined by the presence of 18:1n-7 and 20:1n-9 constituting around 8% and 1% of the total fatty acids, respectively. The role of pike Elovl5 in the biosynthesis of LC-PUFA was investigated by culturing yeast transformed with pYES2-Elovl5 in the presence of C18 (18:3n-3, 18:4n-3, 18:2n-6, 18:3n-6), C20 (20:5n-3, 20:4n-6), or C22 (22:5n-3, 22:4n-6) PUFA substrates. Gas chromatography analysis demonstrated that the yeast transformed with empty pYES2 (control) did not have the ability to elongate LC-PUFA due to a lack of PUFA elongase activity [32]. However, in the presence of pike Elovl5 the C18 and C20 PUFA substrates were efficiently elongated to longer products (Figure 4), whereas C22 PUFA were elongated to a much lower extent not exceeding 4% conversion (Table 1). These results confirmed that pike Elovl5 is involved in the synthesis of LC-PUFA, and presents similar specificities to that described for both salmon Elovl5 paralogs. It was noteworthy that pike Elovl5 was able to convert 18:3n-3 and 18:2n-6 to 20:3n-3 and 20:2n-6, respectively, intermediates in the alternative (Δ8) pathway for the biosynthesis of EPA and ARA [33], through subsequent consecutive desaturations by Δ6Fad (Δ8 activity) to 20:4n-3 and 20:3n-6, and then Δ5Fad to EPA and ARA. However, Elovl5 showed higher activity towards the elongation of 18:4n-3 and 18:3n-6 with over 70% of each PUFA converted to C20 products, whereas 18:3n-3 and 18:2n-6 were elongated less efficiently with around 43% and 28% converted, respectively (Table 1).

**Table 1:** Functional characterisation of Northern pike Elovl5 elongase, and its role in the biosynthesis of long-chain polyunsaturated fatty acids (LC-PUFA)

**PUFA substrate**	**Product**	**Conversion (%)**			**Activity**
		***E. lucius***	***S. salar***	***S. salar***	
		**Elovl5**	**Elovl5a**	**Elovl5b**	
18:3n-3*	20:3n-3	39	-	-	C18 → 20
	22:3n-3	4	-	-	C20 → 22
	Total	43	-	-	
18:4n-3*	20:4n-3	67	56	58	C18 → 20
	22:4n-3	5	7	4	C20 → 22
	Total	72	63	62	
20:5n-3*	22:5n-3	67	36	68	C20 → 22
	24:5n-3	5	1	1	C22 → 24
	Total	72	37	69	
22:5n-3*	24:5n-3	4	1	1	C22 → 24
18:2n-6*	20:2n-6	26	-	-	C18 → 20
	22:2n-6	2	-	-	C20 → 22
	Total	28	-	-	
18:3n-6*	20:3n-6	67	43	65	C18 → 20
	22:3n-6	7	5	6	C20 → 22
	Total	74	48	71	
20:4n-6*	22:4n-6	35	23	48	C20 → 22
	24:4n-6	1	1	1	C22 → 24
	Total	36	24	49	
22:4n-6*	24:4n-6	1	1	1	C22 → 24

Data are expressed as percentage of PUFA converted to elongated products at each step of the pathway and overall when yeast were transformed with pYES2-Elovl5 construct and incubated in presence of C18, C20 or C22 substrates. Atlantic salmon data extracted from [25].

### Tissue distribution of pike Elovl5

**Figure 5:**
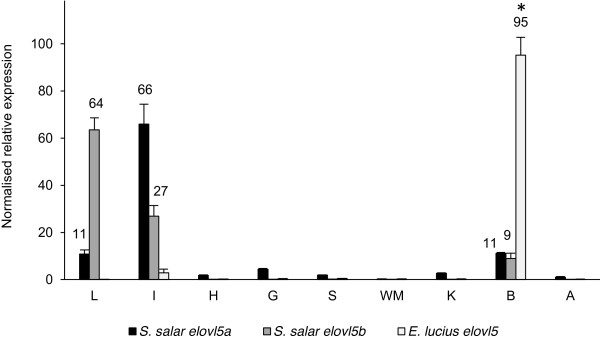
**The tissue distribution of *elovl5 *transcripts (mRNA) in Northern pike and Atlantic salmon.** The relative number of the putative pike *elovl5* transcripts obtained by qPCR were normalised by the geometric mean of the relative copy numbers of the reference genes (*elf-1α* and 18S rRNA). Results are means (± SE) of analysis of three individual pike, and expressed as the percentage relative to the sum of expression across all tissues tested. Normalised expression values of salmon *elovl5a* and *elovl5b* reported in [25] were treated in the same way. * *P* ≤ 0.05 as determined by one-way ANOVA and Tukey’s multiple comparison tests. **L**, liver; **I**, intestine; **H**, heart; **G**, gill; **S**, spleen; **WM**, white muscle; **K**, kidney; **B**, brain; **A**, adipose.

The tissue distribution of pike Elovl5 mRNA transcripts was determined by real-time qPCR. For comparison, the normalised expression values of salmon *elovl5a* and *elovl5b* reported in [25] were treated in the same way and plotted on the same graph. Results indicate the pike *elovl5* was expressed significantly higher in brain (*P* < 0.05) (Figure 5). Thus, compared to expression in liver, expression of *elovl5* in brain was 1000-fold greater, and up to 30-fold higher than intestine. The expression levels in spleen, gill, kidney, white muscle, heart and adipose tissue were negligible, with liver exhibiting the lowest expression. From Figure 5 it is clear that the salmon *elovl5* genes are expressed in a very different pattern, with expression predominating in liver and intestine.

### Segregation analysis and mapping of salmon *elovl5* duplicated loci

The amplicons derived from the *elovl5*-linked microsatellite primer sets resolved clearly and consistently. In both cases allelic size variants, consistent with amplification of a single discrete locus, were detected. Among the pedigree panel parental samples, 12 different alleles were observed for *MS_ elovl5a* and four alleles for *MS_elovl5b.* Joint segregation analysis of the two panels (Br5 and Br5/2), informative for sire based linkage only, did not detect linkage between the two loci (H_o_ = independent assortment; *P* = 0.78 and 0.11 for Br5 and Br5/2, respectively). Genetic mapping of the two *elovl5* loci in the SalMap family (Br5) confirmed the Atlantic salmon paralogs to be located in distinct linkage groups (LG): *elovl5b* on LG 5, and *elovl5a* on LG 33 (LOD scores > 3.5).

## Discussion

The primary aim of this study was to characterise Northern pike Elovl5, a critical enzyme of LC-PUFA biosynthesis in vertebrates. The precise reasons for undertaking the work were ultimately to gain understanding of the evolutionary and ecological adaptations of salmonids. Phylogenetic evidence indicates that esocids are the nearest living relatives of salmonids, having diverged at some point prior to a WGD event in the common ancestor of all salmonids between 25 and 100 mya [1,2]. As WGD has been widely suggested as a major enabling event in evolutionary innovation [34], comparison of single preduplicated genes in pike with their duplicated paralogs in Atlantic salmon has the potential to shed light on evolutionary mechanisms and adaptation in salmonids. The genes of the LC-PUFA biosynthetic pathway are interesting candidates for studies of this type because both genetic and biochemical evidence suggests that salmonids have a higher LC-PUFA biosynthetic capacity than many other fish species [11,20]. Atlantic salmon have duplicated genes for both desaturases and elongases of fatty acids and, in the case of desaturases, duplicates appear to have diverged and neo- or subfunctionalised to provide enzymatic activities for the entire LC-PUFA pathway [26,27]. In contrast, other fish species, particularly marine carnivorous species, are incapable of endogenous production of significant amounts of LC-PUFA because they lack critical genes of the biosynthetic pathway [17,25,35]. It has been suggested that this enhancement of LC-PUFA capacity in salmonids has been a factor in their success in colonising relatively nutrient poor freshwater environments [27]. Salmonids can thrive, and are often the only fish group, in flowing bodies of water, which are reliant on allochthonous terrestrial inputs as a major source of energy. Terrestrial inputs whether directly from leaf litter, or from insect species are poor sources of LC-PUFA, especially DHA, in contrast with food sources in marine or eutrophic freshwater environments which are underpinned by blooms of phytoplankton rich in LC-PUFA [17,20,35,36]. Despite the fact that the tissues of freshwater and marine fish are generally rich in C20 and C22 LC-PUFA, the strategies they utilise to fulfil such requirements vary depending on the species and the dietary input. Northern pike is a strictly freshwater species, whose distribution overlaps that of salmonids, and which shares a relatively recent common ancestor with salmonids. However, pike differ from salmonids in exhibiting a far more piscivorous feeding behaviour. Hence, our particular interest in LC-PUFA biosynthetic genes in this species in comparison to those in Atlantic salmon.

Genetic linkage analyses established that the Atlantic salmon *elovl5* duplicates are located on different linkage groups: *elovl5a* on LG 33, and *elovl5b* on LG 5. Cytogenetic mapping using fluorescence *in situ* hybridisation has assigned salmon LG 33 and LG 5 to chromosomes 28 and 13, respectively [37,38], both of which are acrocentric. While our data clearly show that the two *elovl5* loci are not physically linked, in a recent analysis [39] the Atlantic salmon chromosomes ssa28 and ssa13 were not homeologous and it is therefore not possible to conclude that the salmon *elovl5* paralogous genes are the result of a WGD. It is also possible that this duplication is unique to Atlantic salmon, since no clear evidence of duplicated *elovl5* genes in other salmonids exist in the current sequence databases. However, compared to all other salmonids which have c. 100 chromosome arms, Atlantic salmon is unique in possessing only 72–74 chromosome arms, believed to be the result of species-specific tandem fusions and other rearrangements [38]. Furthermore, linkage maps show [39] that salmon chromosome ssa13 has homeologous regions on at least three other salmon chromosomes, and shares syntenic blocks with at least four separate chromosomes from the diploid stickleback (*Gasterosteus aculeatus*). Thus, given the complexity of the Atlantic salmon genome, it would be premature to reject a WGD origin for the two salmon *elovl5* loci.

Phylogenetic analysis confirmed the basal nature of Northern pike within the protacanthopterigyans. Analysis of the rates of nucleotide substitution in the Atlantic salmon Elovl5 paralogous genes indicated that they are under an evolutionary regime of purifying selective pressure (ω < 1), and are currently evolving at comparable evolutionary rates at the protein level, thus supporting the idea that both duplicates are physiologically required, and have the same biochemical activity as the pike Elovl5. In a larger phylogenetic study, [10] performed pairwise dN/dS analyses on 408 sets of duplicated salmon genes using a preduplicated set of Northern pike orthologs as outgroups, and similarly concluded that salmon paralogs were predominantly exposed to purifying selection, although some loci may be showing a relaxation of selective pressure suggesting that evolution was acting asymmetrically on some paralogs due to reduced constraints. A closer inspection of the dN/dS along the coding sequences of vertebrate *elovl5* suggested the accommodation of a major catalytic site where stronger functional constraints seemed to have acted against the retention of nonsynonymous mutations that would compromise the performance of the enzyme, or impair its activity [40,41].

Here we also tested for functional similarity of pike Elovl5 to salmon paralogs by heterologous expression of the pike enzyme in yeast. Supporting the phylogenetic results, the activity of the pike Elovl5 was indistinguishable from previous assays of the salmon paralogs [25]. Pike Elovl5 was able to lengthen PUFA substrates with chain lengths from C18 to C22. The 18:4n-3, 18:3n-6 and C20 specificity of pike Elovl5 and both salmon Elovl5 paralogs was very similar, and similar to that in other vertebrates [32]. Only very low, residual activity for the production of 24:5n-3 was detected in yeast transformed with the pike elongase when incubated with 22:5n-3, as previously observed in other species, including salmonids [25,32,42]. As 24:5n-3 is an important intermediate in most vertebrates for the biosynthesis of DHA, and in salmonids C22 to C24 activity has been demonstrated in Elovl2 and Elovl4 [25,28], it would be interesting to look for and study these elongase genes in pike.

Until recently the production of LC-PUFA from C18 PUFA was thought to proceed via an alternating desaturation/elongation cycle with the initial step being Δ6 desaturation. However, recent evidence suggests that an alternative pathway is also possible, with the initial step being C18 to C20 elongation, based upon the ability of Δ6 desaturases to also catalyse Δ8 desaturation. For such an alternative loop to exist, elongase activity on LA and ALA is required, and this has been demonstrated in other species [42-44] and, according to results presented here, is also shown by pike Elovl5. Although not yet demonstrated in salmon, we would expect that, given the Δ8 activity of salmon Δ6 desaturases [33], salmon Elovl5 enzymes would also possess this activity.

Although the phylogenetic and functional analyses point towards the maintenance of ancestral activities, the expression profiles indicate functional partitioning in the salmon *elovl5* paralogs. Salmon Elovl5a and Elovl5b have different tissue expression profiles, with Elovl5a being expressed at higher level in intestine and Elovl5b in liver [25]. In addition, the nutritional regulation of mRNA transcription of these genes differs in tissues from fish fed diets containing low levels LC-PUFA [25]. The tissue distribution of pike Elovl5 transcripts showed that the highest expression across the tissues tested was in brain, whereas most other tissues, including liver and intestine, showed very low expression in comparison. In contrast, salmon Elovl5 transcripts were predominantly expressed in liver and intestine, with much lower expression in brain, and even less in other tissues [25]. The pattern of pike Elovl5 tissue expression closely resembles the pattern of LC-PUFA biosynthetic gene expression in carnivorous marine fish, where expression and activity is low in liver and intestine, but high in the brain [45]. Brain, as with all neural tissues, has a high LC-PUFA level [17,46] but, as yet, mechanisms for the biosynthesis, or the transport of fatty acids to the brain are not fully understood [47]. Mammalian brain is only capable of biosynthesising a restricted set of fatty acids [48] and it is clear that fatty acid uptake in the brain is different to uptake in most other tissues, probably due the requirement for passage across the blood–brain barrier. Radiolabeled PUFA injected intraperitoneally in Northern pike could be detected in high concentrations throughout the body, with the exception of brain, which consistently contained the lowest amounts of injected PUFA [23]. Currently, most studies support an energy-dependant mechanism that facilitates and regulates fatty acid uptake influenced by chain length and degree of unsaturation [49,50]. The gene expression results from pike and other carnivorous fish suggest that brain may have endogenous biosynthetic machinery for LC-PUFA to supply the high requirements of this tissue, whereas low expression in liver and intestine indicate a reduced requirement for LC-PUFA, or at least DHA, in these tissues. Although low levels of LC-PUFA biosynthesis have been detected in isolated pike hepatocytes [24], experiments *in vivo* failed to find evidence of significant conversion in liver [23]. In salmon, although Elovl5 and other LC-PUFA biosynthetic genes are expressed in brain, the highest expression levels are in liver and intestine, the main tissues responsible for dietary fatty acid uptake, biosynthesis and distribution [17,25]. Salmonid liver has a comparatively high capacity for biosynthesising LC-PUFA such as EPA and DHA [51], whereas marine carnivorous fish, such as sea bass (*Dicentrarchus labrax*) have negligible capacity depending on dietary LC-PUFA [52].

This above discussion should be qualified by noting that studies on various salmonids and other freshwater species fed with artificial diets with varying LC-PUFA contents and compositions have shown that hepatic desaturase and elongase enzymes exhibited higher expression when the amount of dietary LC-PUFA decreased and LA and/or ALA increased [11,20,25,51]. Although, it is possible that LC-PUFA biosynthetic enzymes in liver are also under nutritional regulation in pike, the fish in the present study were obtained from wild stock and thus essentially piscivorous and so would be equivalent to salmon fed diets containing fish oil (i.e. high in LC-PUFA) [25].

## Conclusions

Northern pike possess an active Elovl5 gene, which is homologous to the duplicated salmon *elovl5a* and *elovl5b* genes. There is no evidence of positive (diversifying) selection acting on the salmon genes, or on the pike gene. On the contrary there is evidence of purifying selection maintaining the activity of all of these genes under functional constraints at the protein level. Supporting this, the enzymatic activities of the pike and the two salmon enzymes are indistinguishable. However, a sharply contrasting expression profile was noted between pike Elovl5 and salmon Elovl5 paralogs. Pike elovl5 expression was restricted to brain, in common with other carnivorous fish, whereas salmon have highest expression in liver and intestine. This may be the result of adaptations; salmon to a diet in freshwater relatively poor in LC-PUFA, while pike have a highly piscivorous diet containing higher levels of LC-PUFA. Future studies on the possible diversification and/or neo-or subfunctionalisation of the duplicated salmon genes should focus on mechanisms of gene expression and transcriptional regulation.

## Methods

### Biological samples

Wild Northern pike were caught in Airthrey Loch, Stirling (Scotland). The individuals were euthanised humanely, immediately and rapidly dissected and the tissue samples stored in RNAlater® (Invitrogen, Paisley, UK) at room temperature until tissue homogenisation was performed 24 h later. Fin clips were obtained and stored in 70% ethanol for genomic DNA extraction. Pike were obtained conforming to local ethical regulations regarding field studies on wild animals, and with the permission of the local fisheries managers.

### Molecular cloning of pike Elovl5 cDNA

Pike Elovl5 cDNA was cloned via RNA extraction, cDNA synthesis and a variety of PCR techniques using the oligonucleotide primers listed in Table 2. Briefly, pike intestinal tissue was used as a source of total RNA, isolated by guanidinium/phenol extraction procedure (TriReagent, Sigma, Poole, UK). Total RNA was transcribed to cDNA using MMLV reverse transcriptase (ImProm-II, Promega) primed with oligo(dT) and random hexamers. The nucleotide sequences of salmon [GenBank:AY170327] and [GenBank:FJ237531], zebrafish [GenBank:NM_200453], Atlantic bluefin tuna [GenBank:HQ214237] and European seabass [GenBank:FR717358.1] Elovl5 cDNA were aligned, and highly conserved regions identified for the design of oligonucleotide primers. A pike Elovl5 partial cDNA was then obtained using a nested PCR design. To obtain the full-length cDNA, rapid amplification of cDNA ends (RACE) with nested Elovl5-specific primers and the SMART 3’ and 5’ oligonucleotide primers (SMART RACE cDNA Amplification Kit, Clontech) was performed, using TaKaRa polymerase (*TaKaRa LA Taq* Hot Start Version, Takara Bio Inc., Clonetech). PCR products were ligated into plasmid pCR2.1 (TA Cloning Kit, Invitrogen), sequenced and data assembled using SeqMan (SeqMan II, Lasergene DNASTAR) to determine the full-length cDNA sequence of pike *elovl5*. Finally, the entire coding sequence of pike *elovl5* was amplified from brain cDNA using the high fidelity Pfu Turbo DNA polymerase (Stratagene, Agilent Technologies, Cheshire, UK) by performing a nested PCR. The first PCR was performed with primers designed in the untranslated regions (UTR), PIKE5OUTF and PIKE5OUTR (Table 2). PCR conditions consisted of an initial denaturing step at 95°C for 1 min, followed by 30 cycles of denaturation at 95°C for 45 s, annealing at 60°C for 45 s, and extension at 72°C for 2 min and a final extension at 72°C for 5 min.

**Table 2:** **Details of primer pairs (restriction sites for *Xba*I and *Bam*HI, and fluorescent CAG and M13R binding sites underlined) used for the cloning of pike Elovl5 ORF in pYES2, qPCR analysis of tissue expression and for the genotyping of Atlantic salmon paralogous genes**

**Species**	**Aim**	**Transcript**	**Primer**	**Primer sequence**	**Amplicon size**	**Ta**	**Accession no.**
*E. lucius*	*elovl5* cDNA cloning	Elovl5	UNIE5OUTF	5’- ATGGATGGGTCCCAGAGA -3’	437 bp	55°C	JX272634^a^
			UNIE5OUTR	5’- AGTTCATAACGAACCACCAGAT -3’			
			UNIE5INNF	5’- TGGGGCCCAAGTACATGA -3’	388 bp	55°C	
			UNIE5INNR	5’- TGGACGAAGCTGTTAAGGG -3’			
	5’ RACE	Elovl5	PIKE5_5’F	5’- GATGGCAGAGAGCCCATAGT -3’	639 bp	55°C	JX272634^a^
			PIKE5_5’R	5’- CCACACAGCAGACACCATCT -3’	336 bp	55°C	
	3’ RACE	Elovl5	PIKE5_3’F	5’- AGATGGTGTCTGCTGTGTGG -3’	1118 bp	60°C	JX272634^a^
			PIKE5_3’R	5’- ATGCTCAACATCTGGTGGTTT -3’	915 bp	60°C	
	ORF cloning	Elovl5	PIKE5OUTF	5’- GCCCAGGTTCGCATCACCCAG -3’	1337 bp	60°C	JX272634^a^
			PIKE5OUTR	5’- ATTCCGGGGGTCATTTGAGATAGACG -3’			
			PIKE5ORF_F	5’- CCCGAGCTCGGATCCAAATGGAGACTTTTAATCAGAGACTTAACACC -3’	922 bp	60°C	
			PIKE5ORF_R	5’- GGGTCTAGACTCGAGCTTCAGTCCGCCCTCACTTTCCT -3’			
	qPCR	18S	qPCRp18SF	5’- TTCGAATGTCTGCCCTATCAAC -3’	128 bp	55°C	Contig2237^b^
			qPCRp18SR	5’- CCTTCCTTGGATGTGGTAGC -3’			
		Elovl5	qPCRpELO5F	5’- CCTTTGCACTGACCGTGATA -3’	195 bp	56°C	JX272634^a^
			qPCRpELO5R	5’- GCGTGTCCTGGCAGTAGAA -3’			
		Ef1-a1	qPCRpEF1F	5’- AAGATCGACCATCGTTCTGG -3’	209 bp	55°C	GH265867^a^
			qPCRpEF1R	5’- CTGGCAGCCTTCTTATCGAC -3’			
*S. salar*	*Genotyping*	Elovl5a	*MS_elovl5a*_F	5’- ACAATTGCCATTTTTGCAGATAGC -3’	175-231 bp^c^	58°C	AGKD01037727^a^
			*MS_elovl5a*_M13R	5’- GGATAACAATTTCACACAGGAGCCATTCTTGATCCGCTTAT -3’			
		Elovl5b	*MS_elovl5b*_CAG	5’- CAGTCGGGCGTCATCAAGCCCGATATGATATTACCGTATT -3’	172-181 bp^c^	56°C	AGKD01030045^a^
			*MS_elovl5b*_R	5’- GTAAAATGGTGACTTTGGGTTCAG -3’			

^a^ GenBank [http://www.ncbi.nlm.nih.gov/].

^b^ University of Virginia, Centre for Medical Research [http://lucy.ceh.uvic.ca/contigs/cbr_contig_viewer.py].

^c^ Scored from 10 parental samples.

### Sequence and phylogenetic analyses

The amino acid (AA) sequence deduced from pike *elovl5* cDNA was aligned with salmonid orthologs, including Atlantic salmon, rainbow trout (*Oncorhynchus mykiss*, [GenBank:NM_001124636.1]), and masu salmon (*Oncorhynchus masou,* [GenBank:DQ067616.1]) using the BLOSUM62 matrix (BioEdit 7.1.3, Tom Hall, Ibis Biosciences, Abbott Laboratories). Further global pairwise alignments of corresponding DNA sequences and those of other vertebrate *elovl5* genes were performed using the Needleman-Wunsch algorithm [http://www.ebi.ac.uk/Tools/psa/emboss_needle/]. A phylogenetic tree was constructed on the basis of the nucleotide sequence alignment between pike *elovl5* coding sequence, and other vertebrate *elovl5*-like ORF in order to take account of single nucleotide substitutions. The 16 vertebrate *elovl*5 sequences used to construct the phylogenetic tree constitute the species that were considered for further analyses. The tree was rooted to *Mortierella alpina* PUFA elongase [GenBank:AF206662] as the outgroup species applying the Neighbour Joining algorithm [53], and the maximum composite likelihood (ML) model using MEGA4 [54]. The bootstrap test was conducted by burning 10,000 trees. Northern pike Elovl5 membrane topology was predicted using PredictProtein tool [http://www.predictprotein.org], which applies the hydrophobicity PHD method (HeiDelberg) with an expected average accuracy of > 98%.

### Tests of selection

The influence of selection on Elovl5 genes in pike and salmon was assessed by determining the rates of nonsynonymous substitutions (dN) versus synonymous substitutions (dS) per site in the coding sequence of pike Elovl5, and vertebrate orthologs. For a nucleotide change in a single codon, a synonymous substitution does not result in a change of the amino acid that is specified, whereas a nonsynonymous substitution does. The “acceptance rate” (ω = dN/dS) termed by [55] is related to evolutionary constraints at the protein level. A value of ω > 1 indicates evidence of Darwinian positive selection whereas ω < 1 suggests negative (purifying) selection, and ω = 1 neutral selection [56].

For greater confidence, a variety of stochastic algorithms were used to assess the dN/dS ratio based on codon-aligned nucleotide sequences. SNAP [57] employs the Nei and Gojobori method [58] incorporating a statistic that allows a more accurate estimation of the variances and covariances of dS and dN [59]. This method allows the use of a best estimate of an ancestral sequence to perform pairwise comparisons between the predicted ancestor and the rest of the sequences, providing an overall ω for the sequences analysed. Similarly, Datamonkey [http://www.datamonkey.org/] is a publically available set of tools implemented by HyPhy package that include robust maximum likelihood approaches to determine the total species dN/dS ratio. The GA-branch analysis (HKY85 model) [60] is based on a genetic algorithm approach that assigns rate classes of ω to lineages in a phylogenetic context. Finally, Tajima’s test implemented by MEGA4 was used to determine the relative rate of nucleotide and amino acid substitution of the salmon Elovl5 paralogs over evolutionary time using pike Elovl5 as an outgroup. This test performs a chi-square (χ^2^) to determine whether the rates of evolution of the two compared sequences are comparable (H_o_), or not (H_A_), thus disrupting the rate of the molecular clock [61].

### Substrate specificity of pike Elovl5

The pike Elovl5 coding sequence was obtained by PCR from full-length cDNA with primers containing restriction sites, PIKE5ORF_F (*Bam* HI) and PIKE5ORF_R (*Xho* I) (Table 2). This fragment and the yeast episomal plasmid pYES2 (Invitrogen) were digested with the corresponding restriction endonucleases (New England BioLabs, Herts, UK), and ligated using T4 DNA Ligase (Bioline, London, UK). *S. cerevisiae* (strain INVSc1) transformation, selection and growth were as described in [28]. The substrate specificity of pike Elovl5 was determined by incubating transformed yeast with one of the following fatty acid substrates: ALA (18:3n-3), LA (18:2n-6), stearidonic acid (18:4n-3, SDA), γ-linolenic acid (18:3n-6, GLA), EPA (20:5n-3), and ARA (20:4n-6). The fatty acid substrates (> 99% pure) and chemicals used to prepare the *S. cerevisiae* minimal medium^-uracil^ were purchased from Sigma Chemical Co. Ltd. Yeast transformed with pYES2 containing no insert were cultured under the same conditions described above and used as control treatment. Yeast fatty acid analysis was as described in [28].

### Tissue distribution of pike Elovl5

Tissue expression of Elovl5 was determined by quantitative real-time PCR (qPCR). Total RNA was extracted from brain, intestine, liver, adipose tissue, white muscle, kidney, spleen, heart and gill from juvenile Northern pike (n = 3) using organic solvent Tri Reagent (Sigma). RNA quality and quantity were assessed by electrophoresis and spectrophotometry (Nanodrop 1000, Thermo Scientific, Wilmington, USA). One microgram of DNase (DNA-free kit, Ambion, Applied Biosystems, Warrington, UK) treated total RNA was reverse transcribed into cDNA using SuperScriptTM III Reverse Transcriptase (Invitrogen) and primed with 5 μM of oligo(dT) and 15 μM of random hexamers (AB Applied Biosystems). Following primer annealing at 25°C for 5 min, and cDNA synthesis at 55°C for 1 h, reactions were stopped by heating at 70°C for 15 min, and cDNA was diluted 5-fold with nuclease-free water. The *elovl5* qPCR primers were designed to anneal to each of two predicted exons spanning an intron, whereas the *ef1-α1* (reference gene) primer pair was designed in a region corresponding to the coding sequence. Table 2 shows the sequence of the primers used, their specific annealing temperatures, the size of fragments produced, and the reference sequences used for primer design, using the Primer3 software [62]. qPCR analyses were performed using a Mastercycler RealPlex^2^ (Eppendorf, Stevenage, UK) in a final volume of 20 μl containing 5 μl of diluted cDNA (1/20 for *ef1-α1* and *elovl5,* and 1/5,000 for 18S rRNA), 0.5 μM of each primer, and 10 μl of SensiMix SYBR No-ROX (Bioline). Amplifications were carried out with a systematic negative control (NTC, no template control) containing no cDNA. Standard curves with dilutions: 1/5, 1/10, 1/20, 1/50, 1/100, 1/200, and 1/500 were prepared for *ef1-α1* and *elovl5*, whereas dilutions ranging from 1/10 to 1/1,000,000 were made up for 18S rRNA given its predominant abundance in the total RNA. Thermal cycling was initiated at 95°C for 10 min, followed by 40 cycles: 15 s denaturing step at 95°C, 15 s at the specific primer pair annealing Tm (Table 2), and 15 s extension at 72°C. After the amplification phase, a melting curve from 60°C to 95°C was performed, enabling confirmation of amplification of a single product in each reaction. The qPCR product sizes were determined by agarose gel electrophoresis and their identity confirmed by sequencing. No primer-dimer formation occurred in the NTC.

Normalised expression values were generated by the ΔCt method [63] using the geometric mean of the Ct values of the two reference genes (*ef1-α1* and 18S rRNA). Results were expressed as the percentage of normalised expression relative to the sum of expression across all tissues tested. Normalised expression values of salmon *elovl5a* and *elovl5b* reported in [25] were treated in the same way. Differences in the expression of pike Elovl5 among different tissues were determined by one-way analysis of variance (ANOVA) followed by Tukey’s multiple comparison test at the significance level of *P* ≤ 0.05 (PASWS 18.0, SPSS Inc., Chicago, USA).

### Linkage analysis of duplicated loci in Atlantic salmon

BlastN searches of *elovl5a* and *elovl5b* cDNA sequences ([GenBank:AY170327] and [GenBank:FJ237531], respectively) identified larger contig fragments for both genes [GenBank:AGKD01037727, GenBank: AGKD01030045] within the preliminary Atlantic salmon genome assembly [GenBank:GCA_000233375.1]. RepeatMasker [64] was used to identify potentially informative microsatellite markers within both sequences. A large dinucleotide repeat (CA)_36_ was located 0.5 kb downstream of *elovl5a*, while the closest marker identified for *elovl5b* was a trinucleotide repeat, (TTA)_10,_ situated 13 kb downstream. PrimerSelect software (DNASTAR Inc., USA) was used to design flanking PCR primers for allele detection (Table 2). Fluorescently-labelled tailed primers [65] were used (detailed in Table 2), the allelic products being detected and sized by capillary electrophoresis (CEQ-8800 Genetic Analysis System, Beckman Coulter Inc., USA). The loci were amplified separately, with 0.5 μL of both reactions being pooled later and electrophoresed in duplex. Each reaction (6 μL final volume) comprised 50 ng template DNA, 20 nM specific forward primer (tailed), 300 nM each of specific reverse primer and fluorescently labelled tail primer and 3 μL 2× MyTaq HS mix (a *Taq* polymerase mastermix from Bioline Reagents Ltd, UK). Hot-start PCR was carried out on a T-gradient thermocycler, (Biometra GmbH, Germany) using the following profiles 95°C for 1 min (for Taq activation and initial denaturation), followed by 34 cycles consisting of 95°C for 15 s, annealing at 58°C (*elovl5b*), or 56°C (*elovl5a*) for 30 s, and extension at 72°C for 45 s.

The parents of six available Atlantic salmon pedigree panels were screened for both loci, with two of these (Br5 and Br5/2) being identified as informative for linkage analysis (i.e. at least one parent being heterozygous for both loci). A total of 46 progeny from family Br5 were screened (more than 400 loci having been previously mapped for this panel (SalMap project, [66]), while 31 progeny available for family Br5/2 were genotyped. Joint segregation analysis [67], as implemented in [68] was carried out on both panels, while linkage mapping was performed on the SalMap reference family (Br5) using JoinMap [69].

## Competing interests

The authors declare that they have no competing interests.

## Authors’ contributions

GCA and MJL planned and coordinated the research; GCA performed laboratory analyses and data analysis; JBT conducted the genetic linkage analysis; GCA wrote the first draft of the manuscript, followed by contributions from remaining authors. All authors read and approved the final manuscript.
